# E-tool for mental health prevention: a study of the receptivity and engagement in a large-scale group of subjects

**DOI:** 10.1192/j.eurpsy.2022.1760

**Published:** 2022-09-01

**Authors:** R. Maçorano, F. Canais, H. Ferreira, M. Parreira, M. Ribas

**Affiliations:** 1 Faculty of Sciences of the University of Lisbon, Institute For Biophysics And Biomedical Engineering, Lisboa, Portugal; 2 NeuroGime, Neuropsychology, Braga, Portugal; 3 NeuroGime, Psychology, Braga, Portugal

**Keywords:** GAD, Biofeedback, burnout, prevention

## Abstract

**Introduction:**

Due to the Covid-19 effects, mental health conditions are now, more than ever, affecting our daily lives - both personally and professionally. The average delay between the onset of first symptoms of a mental health disorder and seeking suitable healthcare is 11 years. The WHO states that the only sustainable way to reduce mental healthcare burden is by acting earlier.

**Objectives:**

The aim of this project is to assess the receptivity and engagement of a mobile app for mental health prevention, amongst a large-scale and heterogeneous group of individuals. The main hypothesis under testing is that people are receptive to actively act towards mental health prevention, despite still being a very neglected and stigmatized topic.

**Methods:**

A mobile app for mental health improvement and disease prevention was developed through the digitalization of positive psychology strategies, such as mood tracking, journaling, breathing exercises, among others, which are personalized to the user through biofeedback. The app aims at teaching people how to autonomously cope with mental health conditions, identifying early signs and redirecting them to proper mental health professionals. The app is being released for a population of 35,000 subjects resident in Portugal.

**Results:**

Receptivity and engagement metrics will be assessed on a weekly and monthly basis, for 3 months, segmented by different subject profiles. Mental health metrics will also be assessed, namely anxiety, depression, and burnout levels - using standard psychiatric scales.

**Conclusions:**

We have yet to draw conclusions from the project; however, we aim to achieve first results in due time.

**Disclosure:**

The aim of this research is to assess the receptivity of mental health prevention strategies using technology, namely a mobile app provided by a company.

